# Stabilization of NCM811 cathode interface through macromolecular compound protective film formed by 2,5-bis(2,2,2-trifluoroethoxy)-benzoic acid additive in lithium metal batteries†

**DOI:** 10.1039/d4ra00737a

**Published:** 2024-05-15

**Authors:** Yixin Hou, Daiheng Song, Peiyao Zhang, Bowen Zhang, Ding Dai, Huifeng Tan

**Affiliations:** a Center for Composite Materials, Harbin Institute of Technology Harbin 150001 China tanhf@hit.edu.cn; b National Key Laboratory of Science and Technology on Advanced Composites in Special Environments, Harbin Institute of Technology Harbin 150080 China; c Key Laboratory of Forest Plant Ecology, Ministry of Education, College of Chemistry, Chemical Engineering and Resource Utilization, Northeast Forestry University Harbin 150001 P. R. China daiding@nefu.edu.cn

## Abstract

Lithium metal batteries (LMBs) offer substantial promise for next-generation energy storage owing to lithium metal's low reduction potential (−3.045 V *vs.* the standard hydrogen electrode) and its high specific capacity of 3860 mA h g^−1^. Among various cathode materials in LMBs, LiNi_0.8_Co_0.1_Mn_0.1_O_2_ (NCM811) is extensively employed because of its notably high specific capacity (over 200 mA h g^−1^) and comparatively lower cost. However, structural stress, nickel ions migration, and uneven Li^+^ deposition in NCM811 particles lead to cracking, irreversible decomposition of active substances, and the growth of mossy Li dendrites, causing severe capacity decline and low Coulomb efficiency in LMBs. In this study, we introduce an effective ethoxyl additive, 2,5-bis(2,2,2-trifluoroethoxy)-benzoic acid (2,5BTBA), directly into the carbonate electrolyte. This additive forms a dense and conductive macromolecular protective film on the NCM811 cathode and lithium metal anode during initial cycles, preventing electrode contact with the electrolyte. Consequently, it safeguards the cathode's structural integrity and enables dense lithium deposition. Adding 3 wt% 2,5BTBA, the Li/NCM811 battery retains a high capacity of 150.60 mA h g^−1^ and 89.41% retention after 700 cycles at 0.5C, maintaining an average Coulomb efficiency of 99.13%. This study presents an efficient and straightforward strategy to enhance the capacity retention of LMBs.

## Abstract 2

2,5-Bis(2,2,2-trifluoroethoxy)-benzoic acid forms a protective film of poly(trifluoroethyl acrylate), leading to improved capacity retention.

## Graphical abstract

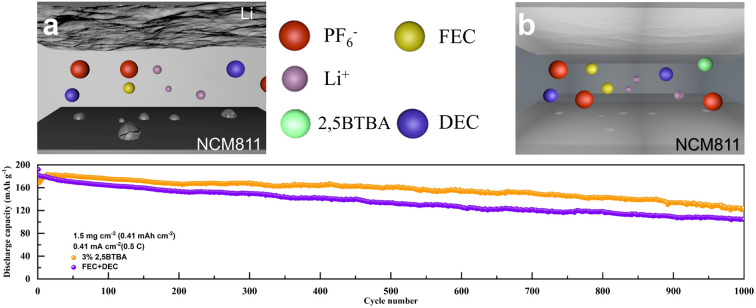

## Introduction

The increasing demand for electric vehicles and portable electronics has made high energy density, long cycle life, and cost-effective batteries a focal point and a driving force in the energy revolution. Lithium-ion batteries (LIBs) comprising graphite anodes and lithium transition metal oxide cathodes have gained significant attention due to their advantages of high energy density, high power density, long cycle life, and absence of memory effect.^1^ LIBs have made substantial progress, particularly in portable electronic equipment, hybrid electric vehicles, and pure electric vehicles. However, they face challenges in surpassing the energy density limit of 300 W h kg^−1^.^2^

The energy density of lithium batteries is projected to reach 500 W h kg^−1^ in the next decade, driven by the swift advancements in electric vehicles and portable electronic devices.^1^ Lithium metal anodes have received extensive attention due to the low density of 0.534 g cm^−3^, the most electronegative potential (−3.04 V *vs.* standard hydrogen electrode) and extremely high capacity (3860 mA h g^−1^).^3,4^ Therefore, LMBs get a tremendous opportunity to meet the goal of energy density.^5,6^ However, LMBs encounter limitations hindering their widespread commercial adoption. For the high chemical activity and electrochemical reactivity, lithium metal readily reacts with the electrolyte, which results in electrolyte depletion, dead lithium accumulation, and uncontrollable dendrite growth, causing potential safety concerns and capacity loss in LMBs.^7–9^

The cathode material is crucial in enhancing the performance of LMBs. Among diverse options, LiNi_0.8_Co_0.1_Mn_0.1_O_2_ (NCM811) garners significant interest owing to its exceptionally high theoretical specific capacity (288 mA h g^−1^) and comparatively lower cost. Nevertheless, based on significant reports detailing the inherent flaws of NCM811, the close similarity in the radii of Li^+^ and Ni^2+^ ions enables dissociative Ni^2+^ to readily occupy Li^+^ sites. This phenomenon compromises the structural stability of NCM811, resulting in irreparable damage to the cathode electrolyte interphase (CEI) and swift capacity degradation.^10–12^ Additionally, the CEI formed on NCM811 is susceptible to cracking as the voltage exceeds 4.0 V in carbonate electrolytes like fluoroethylene carbonate (FEC) and diethyl carbonate (DEC).^9^ Consequently, continual contact between NCM811 particles and the electrolyte induces severe side reactions, causing erosion of active surface sites.^10,13–16^ The breakdown of CEI dramatically reduces the capacity retention of LMBs employing FEC + DEC electrolytes to as low as 80% after fewer than 350 cycles.^17^

Various electrolyte engineering methods have been devised to tackle these challenges. In 2019, Shi's team achieved 93.8% capacity retention after 400 cycles by introducing succinic anhydride to form a conductive solid electrolyte interphase (SEI).^13^ Furthermore, Wang's research group devised a LiI electrolyte additive to fortify both SEI and CEI layers, resulting in extended cycling durability surpassing 600 hours.^18^ Jaumaux *et al.* introduced a deep eutectic solvent based self-healing polymer electrolyte to rise the capacity retention to 86.1% after 200 cycles.^19^ These impactful studies elucidate the broad potential in the field of electrolyte engineering for LMBs. However, these studies have not achieved an exceptionally prolonged cycling life and high-capacity retention with straightforward and cost-effective methodologies.

In this study, we propose an economical and efficient coating strategy by introducing a sacrificial electrolyte additive. This additive decomposes to form a protective film, stabilizing the electrode interfaces. This approach enhances the structural stability of NCM811 and facilitates the uniform deposition of lithium on the Li anode. Direct incorporation of the sacrificial ethoxyl additive, 2,5-bis(2,2,2-trifluoroethoxy)-benzoic acid (2,5BTBA), into the carbonate electrolyte results in the decomposition of the additive, forming a high-polymer film. This film effectively shields the cathode from the electrolyte, thereby impeding parasitic reactions, migration of nickel ions, and degradation of the NCM811 structure. Utilizing 3 wt% 2,5BTBA in the carbonate electrolyte, the Li/NCM811 battery exhibits a remaining capacity of 150.60 mA h g^−1^ after 700 cycles, retaining 89.41% capacity at 0.5C with an average Coulomb efficiency of 99.13%. These results significantly surpass the performance of the Li/NCM811 battery without 2,5BTBA. Our findings introduce a novel approach for developing long-life LMBs through a cost-effective and straightforward method.^4,20–22^

## Results and discussion

### Electrolyte design for high polymer film to protect SEI/CEI

**Fig. 1:**
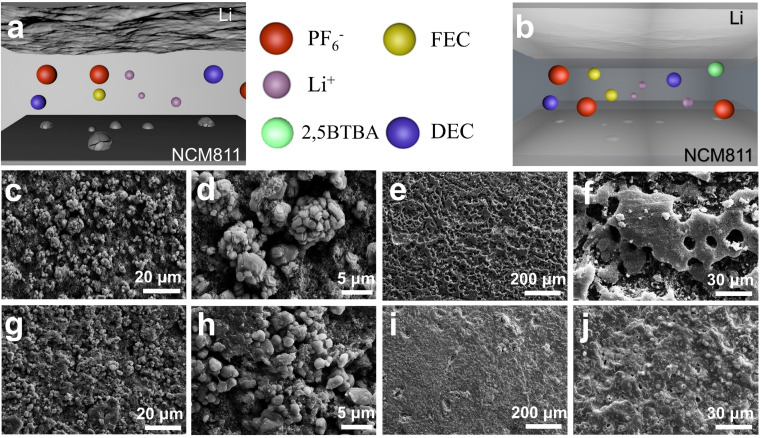
Design strategy of LMBs with 2,5BTBA sacrificial additive. Schematic diagrams of Li/NCM811 batteries without (a) and with (b) 2,5BTBA sacrificial additive; SEM images of cathodes (c, d, g and h) and anodes (e, f, i and j) of Li/NCM811 batteries after 50 cycles at 0.5C, comparing those without (c–f) and with (g–j) 2,5BTBA sacrificial additive.

As illustrated in Fig. 1a, the NCM811 cathode experiences the migration of nickel ions and non-uniform distribution of Li^+^, resulting in structural deterioration of NCM811. This phenomenon causes substantial particle cracking and significant loss of active sites.^23,24^ The resultant damage manifests prominently across the surface of the NCM811 cathode. Conversely, irregular deposition and stripping of lithium ions on the Li anode prompt unregulated lithium dendrite growth.^25,26^ Uncontrolled proliferation of lithium dendrites leads to considerable capacity reduction and diminished Coulomb efficiency.^27,28^

In contrast, upon the introduction of 2,5BTBA, a dense polymer film is generated, acting as a robust shield for both the NCM811 cathode and the Li anode. This film completely envelops the NCM811 cathode and the lithium metal anode, forming a protective layer that prevents further reactions between the electrodes and the carbonate electrolyte, effectively averting cracking of NCM811 particles and dendritic growth of lithium metal (Fig. 1b). Throughout the cycling process, mechanical stress initiates cracking in the NCM811 particles. Furthermore, the dissolution of transition metals exacerbates these cracks, as evidenced by the scanning electron microscopy (SEM) images of the cycled NCM811 cathode under high voltage (Fig. 1c and d). The cracks intensify parasitic reactions between the NCM811 and electrolyte, leading to continuous depletion of active substances and rapid capacity loss.^29^ Additionally, the cycled Li/NCM811 battery lacking 2,5BTBA exhibits characteristic mossy dendrites on the lithium metal anode, evident in Fig. 1e and f. These dendrites result from heterogeneous lithium deposition, causing irreversible electrolyte consumption and subsequent capacity loss.^30,31^ The structural deterioration observed in the NCM811 cathode and the presence of mossy dendrites on the Li anode indicate the instability of the SEI and CEI layers formed in the cycled Li/NCM811 battery without 2,5BTBA. This instability complicates the prevention of parasitic effects that damage active sites, consequently leading to substantial capacity degradation.^32^ Higher magnification SEM images (Fig. S5 and S6†) also confirmed that the cathode surface in batteries with 2,5BTBA was covered with a layer of high polymer (Fig. S5a and c†). This coverage resulted in a smoother cathode surface (Fig. S5b and d†) and fewer cracks in the NCM811 crystal (Fig. S6†).

In contrast, upon decomposition, 2,5BTBA undergoes a vigorous transformation, generating a high-polymer film that envelops both the NCM811 cathode and the lithium metal anode (Fig. 1b). Notably, in the cycled Li/NCM811 battery with 2,5BTBA, evident in Fig. 1g and h, the NCM811 cathode displays a smooth surface without observable cracks. Instead, the surface is covered with a moss-like substance, which constitutes the high-polymer film. This dense and uniform polymer film effectively isolates the electrolyte from the NCM811 cathode while maintaining high lithium ion conductivity.^33–35^ Similarly, due to the protective film, the lithium metal anode exhibits a notably flatter surface, devoid of the typical mossy lithium dendrites, thereby preventing dendritic penetration of the separator and enhancing the safety profile of LMBs (Fig. 1f and j).

### Physicochemical properties of electrolytes and polymer film

**Fig. 2:**
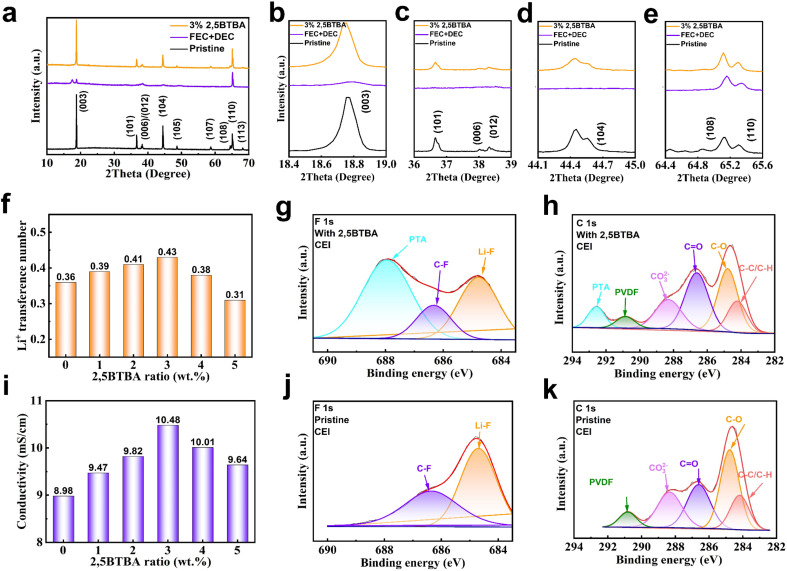
Optimization of the interface between NCM811 cathodes and electrolytes. Comparison of XRD patterns of the NCM811 with 2,5BTBA and without 2,5BTBA after 50 cycles (a–e); composition analysis of F 1s spectra (g and j) of NCM811 in electrolyte without 2,5BTBA (j) and with 2,5BTBA (c), and C 1s spectra (h and k) without 2,5BTBA (g) and with 2,5BTBA (d); Li^+^ transference number (f) and conductivity (i) of Li/NCM811 batteries with different weight ratio of 2,5BTBA additive. All electrodes of XPS and LSV are tested after 50 cycles at 1C.

According to numerous reports, the CEI formed in the carbonate electrolyte and Ni^4+^ environment tends to be unstable and susceptible to degradation during cycling, resulting in structural damage to the NCM811 cathode.^36,37^ To assess the structural integrity of the NCM811 cathode, X-ray diffraction (XRD) analysis was utilized to examine the structural characteristics of NCM811 from batteries both with and without 2,5BTBA after cycling. As depicted in Fig. 2a, the characteristic peaks of NCM811 in batteries lacking 2,5BTBA are notably weak, almost vanishing. Conversely, in the presence of 2,5BTBA, the distinctive diffraction peaks attributed to NCM811 are clearly preserved even after 50 cycles, as the decomposition of 2,5BTBA leads to the formation of a protective polymer film. Notably, these peaks are more pronounced than those observed in NCM811 without 2,5BTBA.

Analysis of the characteristic peaks of (003) and (104) (Fig. 2b and d) reveals significant structural damage to NCM811 after 50 cycles in batteries without 2,5BTBA, owing to the deleterious impact of the electrolyte on NCM811. However, in the presence of the 2,5BTBA additive, the characteristic peaks of (003) and (104) in the cycled Li/NCM811 battery remain nearly unchanged compared to pristine NCM811. Furthermore, in Fig. 2a, it is clearly observable that the XRD peaks of the battery without the addition of 2,5BTBA exhibit a certain degree of splitting, such as the peak of (003). This phenomenon can be attributed to the structural damage, distortion, and irregularities in the NCM811 crystal lattice induced by stress accumulation and nickel ion migration during charge–discharge cycling. Substantial splitting of the amplified (006)/(102) and (108)/(110) peaks is observed in the cycled Li/NCM811 with 2,5BTBA (Fig. 2c and e), signifying the maintenance of the excellent layered structure of the NCM811 cathode.^12,38^ Additionally, the ratio of *I*_(003)_/*I*_(104)_ is able to reflect the degree of cation mixing.^39,40^ This ratio is 2.41 in the cycled Li/NCM811 battery with 2,5BTBA, higher than that of the battery lacking 2,5BTBA (1.644). A higher ratio of *I*_(003)_/*I*_(104)_ signifies reduced cationic mixing, further demonstrating the protective influence of 2,5BTBA on the NCM811 structure.^41^

X-ray Photoelectron Spectroscopy (XPS) analysis was conducted to explore the valence distribution of C and F elements in the cycled cathodes. The high-resolution XPS spectra of C 1s exhibit peaks corresponding to C–C/C–H, C–O, C=O, CO_3_^2−^, and PVDF bonds. Specifically, the C–O bond formation is attributed to the decomposition of FEC and DEC in the electrolyte, whereas the C=O bond represents constituents and serves as a stabilizer of CEI (Fig. 2h and k).^20,42^ The XPS results illustrate that the incorporation of 2,5BTBA leads to a decrease in the strength ratio of the C–O bond while elevating the ratio of the C=O bond strength. This observation suggests that 2,5BTBA effectively mitigates the electrolyte solvent decomposition and enhances the stability of the CEI layer.

Additionally, the XPS analysis results further validate the decomposition of the additive into a polymer film covering the NCM811 and lithium metal surfaces, corroborating the SEM findings. In Fig. 2g and j, distinct peaks appear in both the F 1s and C 1s spectra, identified as the organic high polymer, poly(trifluoroethyl acrylate) (PTA). Given the structural similarity, it is deduced that this polymer is a byproduct of the 2,5BTBA decomposition. The polymer film integrates numerous high-polarity CF_3_ groups into the CEI, ensuring thorough wetting of both the anode and cathode surfaces. This action restrains the migration of Ni^4+^ ions, safeguards the CEI from damage, and facilitates dense and uniform lithium deposition.^43,44^ Furthermore, the F 1s spectra on the lithium metal surface (Fig. S3†) affirm the presence of PTA on the anode. A distinctive peak around 687 eV, corresponding to the PTA peak in Fig. 2g, displays significantly high intensity. Additionally, the prominence of the C=O band peak in the Li/NCM811 battery with 2,5BTBA surpasses that in the group lacking 2,5BTBA, further supporting the existence of PTA.

The linear sweep voltammetry data of those additives has been incorporated into the ESI (Fig. S10†). It is evident that batteries with the inclusion of 2,5BTBA demonstrate higher currents when the voltage reaches 4 V or above. This is attributed to the decomposition of 2,5BTBA under voltage, leading to the production of PTA. In addition, it is noteworthy that the mixed electrolyte of DEC and FEC is incapable of withstanding voltages exceeding 5 V. However, even beyond this threshold, the resulting current remains scarcely above 0.03 mA. Nevertheless, the current rise is insignificantly gradual. The observed peak current surpassing 0.6 mA in batteries augmented with 2,5BTBA significantly eclipses the current in FEC + DEC electrolytes. This suggests that the considerable spike in current may be attributed to the decomposition of 2,5BTBA under the influence of voltage, aligning well with conclusions proposed in the study.

The impedance of the electrolyte was assessed using electrochemical impedance spectroscopy (EIS) testing (Fig. S1 and S2†). Fig. 2i demonstrates that the introduction of 2,5BTBA and the presence of the PTA film resulted in a notable increase in ionic conductivity. The PTA film contributes to achieving a smoother and more compact interface, enhancing the electrolyte's wettability and consequently augmenting battery conductivity.^45,46^ Moreover, the highest conductivity observed at 3 wt% reveals a 17% improvement compared to batteries lacking 2,5BTBA, indicating markedly enhanced conductivity. Furthermore, the lithium-ion transference number in the electrolyte exhibits a 19.4% increase when compared to the electrolyte lacking 2,5BTBA (Fig. 2f).

### Electrochemical behaviors of Li/NCM811 battery with 2,5BTBA electrolyte

**Fig. 3:**
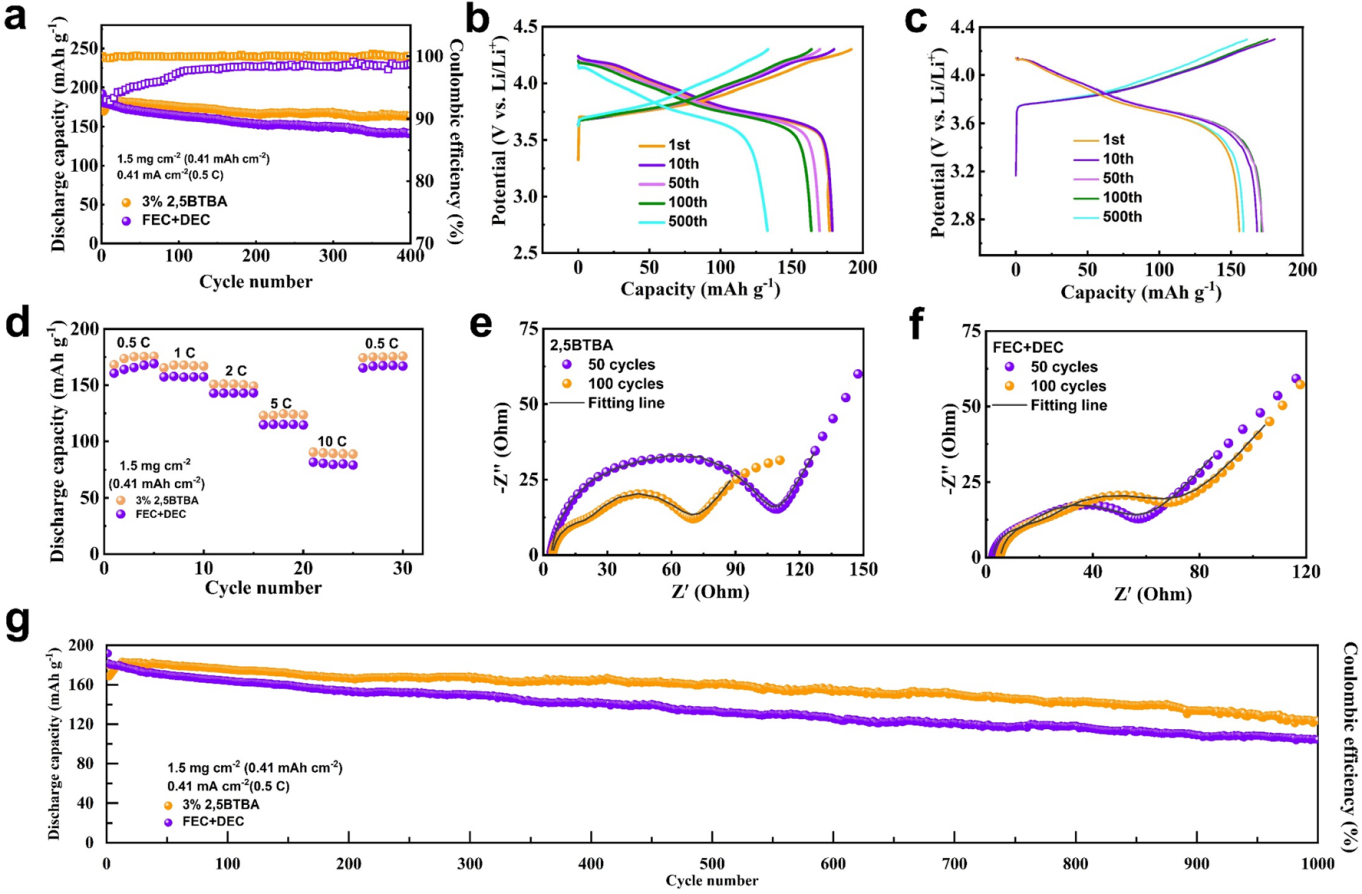
Electrochemical performance improvement assisted by 2,5BTBA. (a) Cycling performance and coulombic efficiency of Li/NCM811 batteries with 2,5BTBA and without 2,5BTBA tested at 25 °C and rate of 0.5C; capacity–voltage curves of Li/NCM811 battery without 2,5BTBA (b) and Li/NCM811 with 2,5BTBA battery (c) tested at 25 °C after different cycles; (d) rate performance of Li/NCM811 batteries without 2,5BTBA and with 2,5BTBA; impedance spectra analyses of Li/NCM811 battery with 2,5BTBA (e) and without 2,5BTBA (f) after different cycles; (g) long cycling performance of Li/NCM811 batteries with 2,5BTBA and without 2,5BTBA tested at 25 °C and rate of 0.5C.

The presence of the PTA film significantly enhances the electrochemical performance of the Li/NCM811 battery with the 2,5BTBA additive. As illustrated in Fig. 3a, the cyclability of the batteries noticeably improves upon the introduction of 2,5BTBA, surpassing the performance of Li/NCM811 batteries lacking this additive. Particularly noteworthy is the achievement of 99% capacity retention after 400 cycles with a 3% weight ratio of 2,5BTBA.

Below 2% weight ratio, the benefits of 2,5BTBA to the batteries are minimal, as insufficient 2,5BTBA fails to form a stable PTA film. Conversely, when the weight ratio exceeds 3%, the advantage over the group without 2,5BTBA diminishes (Fig. S8†). Higher concentrations of 2,5BTBA lead to an excessively thick PTA film, hindering Li^+^ transport. Additionally, concentrations exceeding 5 wt% render 2,5BTBA insoluble in the carbonate electrolyte. The PTA film produced by a 3 wt% concentration of 2,5BTBA maintains a moderate thickness, facilitating Li^+^ transport while effectively preventing parasitic reactions.

Interestingly, an initial rise in capacity by approximately 25 mA h g^−1^ is observed during the first few cycles in the Li/NCM811 battery with 2,5BTBA. Although the initial capacity of the Li/NCM811 battery with 2,5BTBA is slightly lower than the battery without 2,5BTBA, this increase in capacity quickly narrows the gap and even surpasses it within the first 10 cycles. Subsequently, the capacity curve gradually levels off, demonstrating minimal attenuation. This phenomenon is attributed to the formation of the PTA film. During the initial 10 cycles, the reaction of 2,5BTBA causes a slight loss in capacity due to decreased conductivity. This inference is corroborated by the Nyquist plots, revealing decreased impedance in the first 100 cycles in the battery with 2,5BTBA (38.22 Ω at 50 cycles and 14.62 Ω at 100 cycles), whereas increased impedance is observed in the battery without 2,5BTBA (9.365 Ω at 50 cycles and 14.98 Ω at 100 cycles) (Table S1†). However, as the film stabilizes, the interaction between the carbonate electrolyte and cathode is significantly restrained, resulting in the plateauing of the capacity curve. Although the capacity curves of both groups exhibit similar trends, the curve of the 2,5BTBA group is notably smoother, indicating slower capacity decay. Owing to the improved electrolyte wettability and prevention of parasitic reactions, the Li/NCM811 battery with 2,5BTBA achieves 97.8% capacity retention after 300 cycles and 89.4% after 700 cycles. In contrast, the Li/NMCM811 battery without 2,5BTBA reaches only 83.0% capacity retention in just 300 cycles. Additionally, the disparity in cell degradation is further elucidated by the capacity–voltage curves (Fig. 3b and c).^11^ Furthermore, after 1000 cycles, the Li/NCM811 battery with 2,5BTBA maintains a remaining capacity of 123.4 mA h g^−1^ and a capacity retention of 72.65% at 0.5C, boasting an average Coulomb efficiency of 99.13%. Conversely, the battery without 2,5BTBA exhibits a remaining capacity of only 104.33 mA h g^−1^, with the capacity retention dropping to 57.42%.

The PTA film formed by 2,5BTBA also contributes to improved rate performance. A detailed comparison of the rate performance between LMBs without and with 2,5BTBA is presented in Fig. 3c. The battery featuring a 3 wt% 2,5BTBA additive exhibits remarkable specific capacities of 168.14, 165.39, 150.66, 122.98, and 90.55 mA h g^−1^ at rates of 0.5C, 1C, 2C, 5C, and 10C, respectively. This performance enhancement is attributed to the accelerated Li^+^ transport kinetics facilitated by the PTA film. Upon returning to a rate of 0.5C, the discharge capacity registers at 167.00 mA h g^−1^, retaining 99.32% of the initial cycle's capacity. The sustained capacity after reverting to the original current density is due to the stabilizing effect of the PTA film on SEI and CEI. In contrast, the capacities of batteries without 2,5BTBA are notably lower (160.62, 157.39, 142.76, 114.82, and 81.85 mA h g^−1^ at rates of 0.5C, 1C, 2C, 5C, and 10C, respectively), indicating a superior adaptability to high currents exhibited by the 2,5BTBA electrolyte and the resultant PTA film. Simultaneously, Fig. S11† illustrates the rate performance of batteries with varying mass fraction of electrolyte additives. It is observable that the battery with 1 wt% 2,5BTBA exhibits slightly higher rate capability compared to the one without 2,5BTBA, yet slightly lower than the battery with 3 wt% 2,5BTBA. However, the rate performance drastically deteriorates upon the addition of 5 wt% electrolyte additive, rendering it inoperable at a current of 10C. This underscores that the optimal mass fraction for the electrolyte additive is 3%.

Moreover, the application of the 3% 2,5BTBA electrolyte surpasses the performance of state-of-the-art carbonate ether electrolytes in terms of coulombic efficiency, providing further confirmation of its capacity retention capability. Remarkably, the coulombic efficiency of the 3% 2,5BTBA electrolyte remains consistently close to 100% even after more than 750 cycles (Fig. 3g), showcasing the remarkable stability of the enhanced electrolyte's capacity. In comparison, the battery lacking 2,5BTBA displays a less stable coulombic efficiency over the same cycle span.

**Fig. 4:**
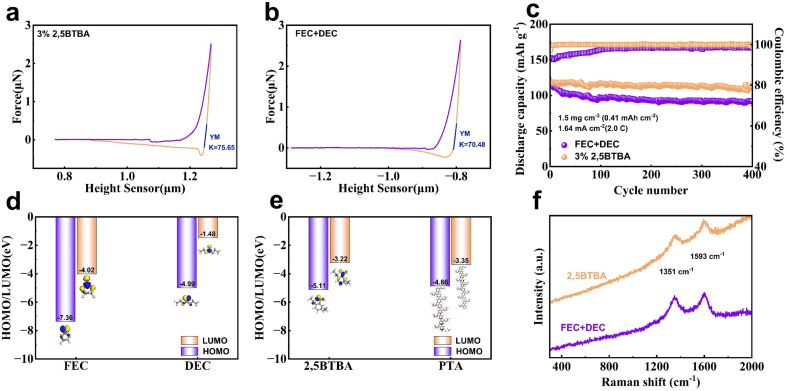
Stability of CEI. Force curve of CEI in batteries with 2,5BTBA (a) and without 2,5BTBA (b) measured by AFM; (c) cycling performance and coulombic efficiency of Li/LFP batteries with 2,5BTBA and without 2,5BTBA tested at 25 °C and rate of 2C; HOMO and LUMO levels of FEC, DEC (d), 2,5BTBA and PTA (e); (f) Raman spectrum of cathodes from battery with 2,5BTBA and without 2,5BTBA.

As depicted in Fig. 4a and b, the force curves measured by atomic force microscope (AFM) indicate that the Young's modulus of the CEI in batteries with 2,5BTBA is approximately 8% higher compared to batteries without 2,5BTBA.^47^ This finding suggests that the formation of PTA contributes to some extent to the enhanced strength of CEI. Furthermore, in Fig. 4d and e, the graphs illustrate the highest occupied molecular orbital (HOMO) energy and the lowest unoccupied molecular orbital (LUMO) energy of organic solvents (FEC, DEC, and 2,5BTBA) and PTA. The HOMO energy of PTA (−4.86 eV) closely resembles that of DEC (−4.99 eV), indicating the high stability of CEI.^48,49^ Additionally, the proximity of the HOMO and LUMO energy levels suggests that 2,5BTBA can readily decompose to form the PTA film.

Further insight into the influence of 2,5BTBA on the positive electrode is obtained through Raman spectra analysis, revealing minimal impact on the NCM811 electrode (Fig. 4f). The spectra exhibit two distinct peaks at 1351 cm^−1^ (D peak) and 1593 cm^−1^ (G peak), characteristic of graphite materials within the cathode structure. The ratio of the area under the D peak to that under the G peak is 0.74 in the battery with 2,5BTBA and 0.81 without 2,5BTBA. A higher ratio signifies a higher defect density within the graphite materials, suggesting the protective influence of 2,5BTBA on the cathode's graphite components.^50,51^

Moreover, 2,5BTBA demonstrates remarkable efficacy in safeguarding diverse cathode materials. When the cathode material is substituted with LiFePO_4_ (LFP), the cycle-specific capacity of the battery with 2,5BTBA remains notably higher compared to the battery without 2,5BTBA (Fig. 4c). After 400 cycles at a rate of 2C, the capacity of batteries with 2,5BTBA remains at 111.43 mA h g^−1^, whereas batteries without 2,5BTBA only retain 91.99 mA h g^−1^.

## Conclusion

The direct addition of the ethoxyl electrolyte additive, 2,5-bis(2,2,2-trifluoroethoxy) benzoic acid (2,5BTBA), into the carbonate electrolyte significantly enhances the performance of Lithium Metal Batteries (LMBs) featuring NCM811 cathodes as well as Li anodes. Upon decomposition, 2,5BTBA forms a dense and conductive protective film, namely poly(trifluoroethyl acrylate), which effectively isolates the carbonate electrolyte from the electrodes and suppressed the degradation of NCM811 crystal and the corrosion of lithium metal surface and the generation of lithium dendrites. This preventive measure mitigates adverse reactions during the charging and discharging cycles. Leveraging 2,5BTBA, LMBs exhibit reduced impedance, demonstrating enhanced capacity at 144.6 mA h g^−1^ and a capacity retention of 87.42% after 750 cycles. In stark contrast, batteries lacking 2,5BTBA only retain 64.5% of their initial capacity after the same cycling duration. Notably, 2,5BTBA exhibits promising compatibility with LFP anodes. This study presents an efficient and straightforward approach to bolstering the capacity retention of LMBs, offering a new perspective in the domain of lithium metal battery electrolyte engineering.

## Conflicts of interest

There are no conflicts to declare.

## Supplementary Material

RA-014-D4RA00737A-s001
