# Supplementary material for: Stabilization of NCM811 cathode interface through macromolecular compound protective film formed by 2,5-bis(2,2,2-trifluoroethoxy)-benzoic acid additive in lithium metal batteries
Source: RSC Adv. 2024 May 15;14(22):15804–11. doi: 10.1039/d4ra00737a (PMC11095362; doi:10.1039/d4ra00737a)
Supplement: RA-014-D4RA00737A-s001 [file RA-014-D4RA00737A-s001.pdf]

Electronic Supplementary Material (ESI) for RSC Advances.
This journal is © The Royal Society of Chemistry 2024

Supporting Information

Stabilization of NCM811 Cathode Interface through Macromolecular Compound Protective Film Formed by 2,5-Bis(2,2,2-trifluoroethoxy)-benzoic Acid Additive in Lithium Metal Batteries

Yixin Hou[1,2,*], Daiheng Song[1,2,*], Peiyao Zhang[1,2], Bowen Zhang[1,2], Ding Dai[3,#] Huifeng Tan[1,2, #]

[1]Center for Composite Materials, Harbin Institute of Technology, Harbin, 150001, China
[2]National Key Laboratory of Science and Technology on Advanced Composites in Special Environments, Harbin Institute of Technology, Harbin 150080, China
[3]Key Laboratory of Forest Plant Ecology, Ministry of Education College of Chemistry, Chemical Engineering and Resource Utilization, Northeast Forestry University, Harbin 150001, P. R. China

---

* Authors contribute equally to this work
[#] Corresponding authors. tanhf@hit.edu.cn (H. Tan), daiding@nefu.edu.cn

Experimental section

Materials

NCM811 (99.9%), binder (PVDF-5130 ,99.9%), and conductive carbon (99.9%) were provided by Guangdong Canrd New Energy Technology Co., Ltd.

2,5-Bis(2,2,2-trifluoroethoxy)-benzoic acid (2,5BTBA, 99.99%) and N-methyl pyrrolidone (NMP, >99.5%) were purchased from Shanghai Aladdin Biochemical Technology Co.

All chemical reagents were analytical grade.

Preparation of 2,5-BTBA electrolytes

A mixed solvent of 1 M $LiPF_6$ in FEC/DEC (v/v=1:1) (provided by Guangdong Canrd New Energy Technology Co., Ltd.) was prepared. For the fabrication of electrolyte with 2,5BTBA, 0.026 g, 0.051 g, 0.078 g, 0.132 g, 0.168 g 2,5BTBA were added into 2.5 mL solvent to make the mass ratios of 2,5BTBA reach 1%, 2%, 3%, 4% and 5%. For the fabrication of electrolyte without 2,5BTBA, the solvent of 1 M $LiPF_6$ in FEC/DEC (v/v=1:1) was directly used.

Preparation of electrodes

For the fabrication of NCM811 cathode, NCM811 (0.6 g), conductive carbon (0.075 g), and binder (PVDF-5130, 0.075 g) were mixed with a mass radio of 8:1:1 in N-methyl pyrrolidone (2.5 mL) and stirred for 24 hours to obtain a uniform slurry. The slurry was evenly coated on aluminum foil for cathode and dried in vacuum at 80 °C

for 12 h. The resulted cathode electrodes were cut into circular sheets with a diameter of 12 mm and the regular loading density of the NMC811 was 1.81-2.37 mg/cm^2.The preparation of LFP cathode is the same as that of NCM811 cathode.

Lithium metal anodes were made of lithium foil (provided by Guangdong Canrd New Energy Technology Co., Ltd.) that cut into 14 mm in argon atmosphere.

Materials characterizations

The surface morphologies of the samples were performed by the scanning electron microscope (SEM, Zeiss Supra, Germany). The valence states of C, F, and Ni elements after cycling were carried out by X-ray photoelectron spectroscopy (XPS, Thermo-Fisher, ESCALAB 250Xi, U.S.A.). Battery disassembling and electrode cutting were carried out and then sealed in the glove box. Then, the sealed sample was transferred into the XPS chamber, taken out rapidly in the chamber and vacuumed immediately to avoid exposure and oxidation in air. All profiles were calibrated at 284.6 eV. X-ray diffractometer (XRD, BRUKER, D8, Germany) and Atomic Force Microscope (AFM, Bruker Dimension Icon, Germany) was used to study the structure of the samples. The material and structure information were analyzed by Raman spectroscopy (Raman, Renishaw inVia, Renishaw, England).

Electrochemical measurements

All electrochemical tests in this work were carried out in a CR2025-type coin cell. The assembling processes were performed in an argon filled glove box (H_2O, $O_2 < 0.01$ ppm). The separators of the cell were commercial Celgard 2500. The amount of the electrolyte was 80 μL. The galvanostatic measurements of LMBs were performed on

the battery testing system (NEWARE, CT-3008), within the voltage range of 2.7-4.3 V (*vs.* Li^+/Li) at a constant temperature of 25 °C. In the first two cycles, all batteries were activated with a low current of 1/15 C, while the following cycles were cycled at a rate of 0.5 C (1 C = 188 mAg^{-1} *vs.* Li^+/Li). Disregarding the first two cycles of activation, the capacity was calculated on the basis of the third cycle. Linear sweep voltammetry (LSV) was tested between 3.0 V and 10.0 V (Shanghai Chenhua, CHI 760e, China). On the same workstation, the Electrochemical Impedance Spectroscopy (EIS) of LMBs at different cycles of 50 and 100 were measured at a frequency of 10 mV (frequency ranging from 100 kHz to 0.1 Hz).

The Li^+ transference numbers for electrolytes were studied with AC impedance and DC polarization analysis. The polarization currents, referred to the initial current (I_0) and steady-state current (I_S) of the Li/Li cell, were obtained with a polarization potential (ΔV) at 10 mV. In addition, the initial and steady-state interfacial resistances (R_O and R_S) of Li/electrolyte were determined by impedance measurements before and after potentiostatic polarization. The impedance measurement is performed at an open circuit potential in the frequency range from 0.10 Hz to 1.0 MHz. t_{Li+} was calculated based on Bruce Vincent Evans equation (Eq 1):

$$t_{Li^+} = \frac{I_S(\Delta V - I_O R_O)}{I_O(\Delta V - I_S R_S)} \tag{1}$$

The ionic conductivity was calculated based on the equation (Eq. 2):

$$\sigma_{electrolyte} = \frac{L_{separator}}{SR_{electrolyte}} \tag{2}$$

where $L_{separator}$ is the thickness of the separator, S stands for the area of the electrode

and $R_{electrolyte}$ represents the Ohmic resistance of the electrolyte, which was tested at an open-circuit potential in a frequency range from 0.10 Hz to 1.0 MHz.

The improved value of interface ion conductivity was calculated based on the equation (Eqs. 3-5):

$$\sigma_{electrode\ and\ interface} = \frac{L_{electrode\ and\ interface}}{SR_{electrode\ and\ interface}} \tag{3}$$

$$R_{electrode\ and\ interface} = R - R_{electrolyte} \tag{4}$$

$$\Delta\sigma_{interface} = \Delta\sigma_{electrode\ and\ interface} \tag{5}$$

where $R_{electrolyte}$, $R_{electrode\ and\ interface}$ and R represent the resistance of electrolyte, the resistance of electrode as well as interface and total resistance, respectively. $\sigma_{electrode\ and\ interface}$ represents the ion conductivity of the interface and electrode. $L_{electrode\ and\ interface}$ represents the thickness of the electrode and interface. Since the interface thickness is small enough relative to the electrode thickness, it is assumed that $L_{electrode\ and\ interface}=L_{electrode}$. Since the electrodes used in the two electrolyte systems are the same, $\Delta\sigma_{interface} = \Delta\sigma_{electrode\ and\ interface}$.

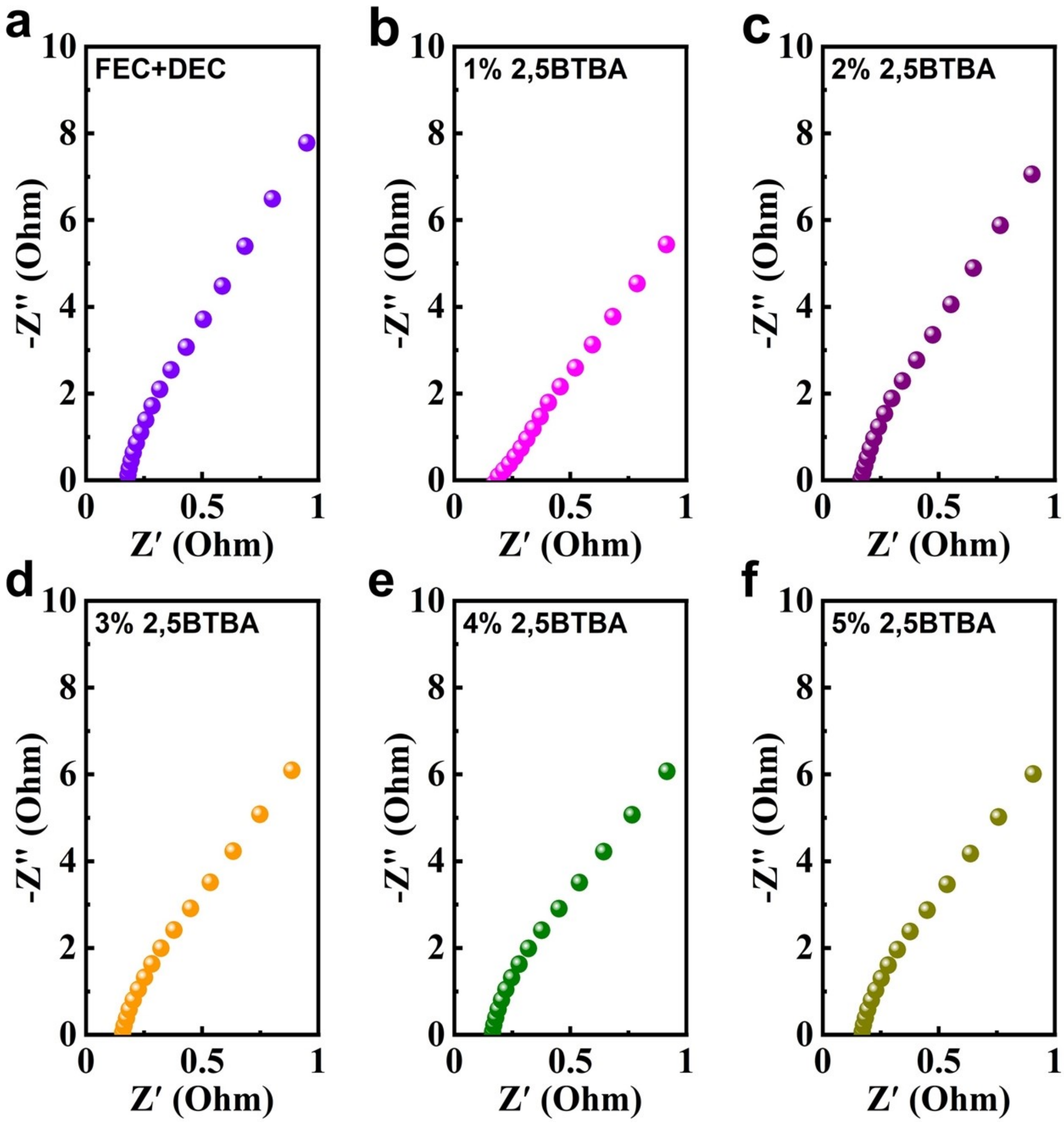

Figure S1. Conductivity of electrolytes without 2,5BTBA (**a**) and with 2,5BTBA of different weight ratio of 1% (**b**), 2% (**c**), 3% (**d**), 4% (**e**) and 5% (**f**).

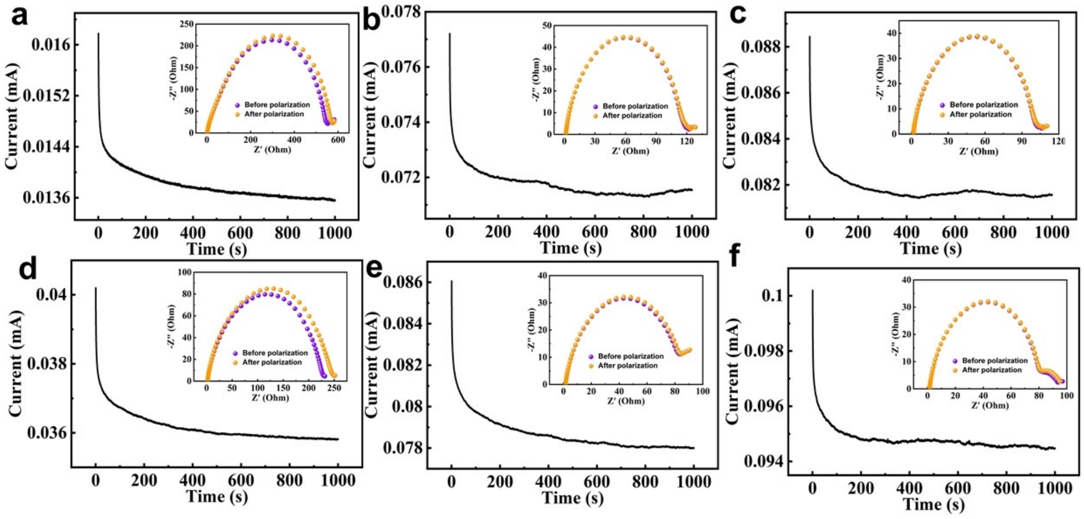

Figure S2. Conductivity before and after polarization and polarization curve with the content of 0.40 (**a**), 0.51 (**b**), 0.58 (**c**), 0.65 (**d**), 0.72 (**e**) and 0.78 (**f**).

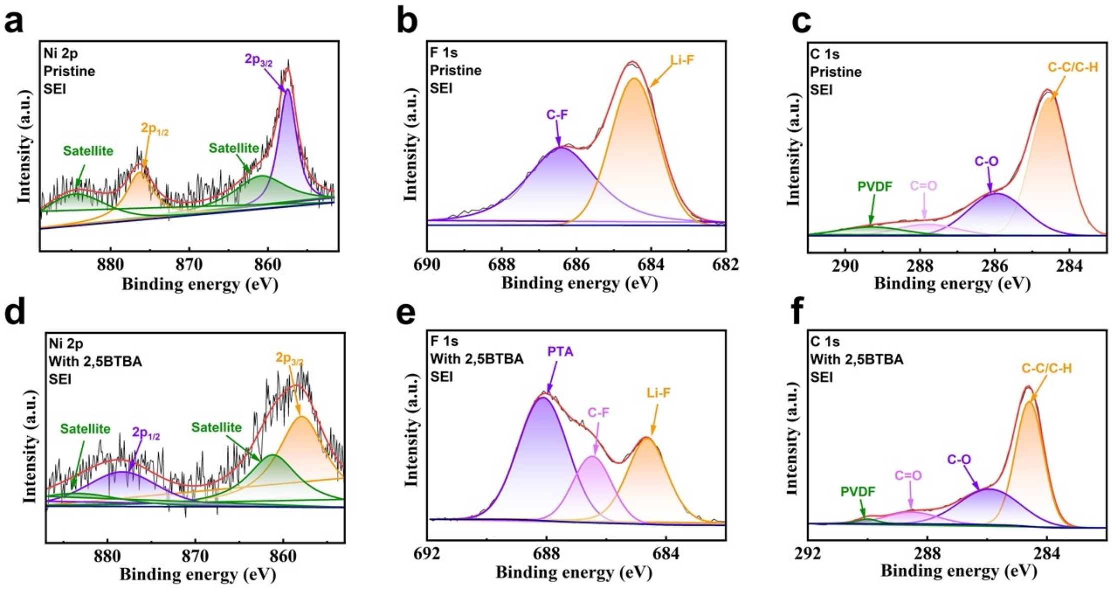

Figure S3. Composition analysis of Ni 2p spectra (**a**, **d**), F 1s spectra (**b**, **e**) and C 1sspectra (**c**, **f**) of Li anodes without 2,5BTBA (**a**, **b** and **c**) and with 3%.wt 2,5BTBA after 50 cycles (**d**, **e** and **f**).

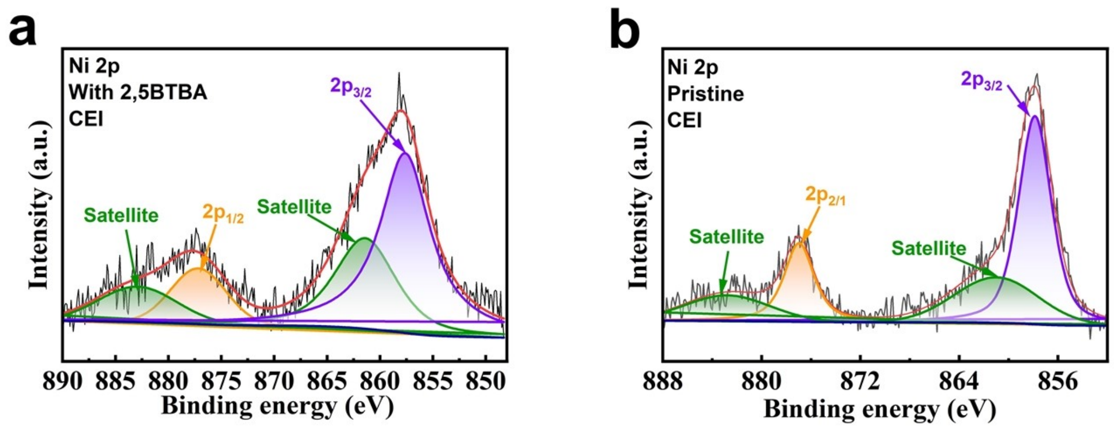

Figure S4. Composition analysis of Ni 2p spectra of cathodes without 2,5BTBA (**b**) and with 2,5BTBA (**a**) after 50 cycles.

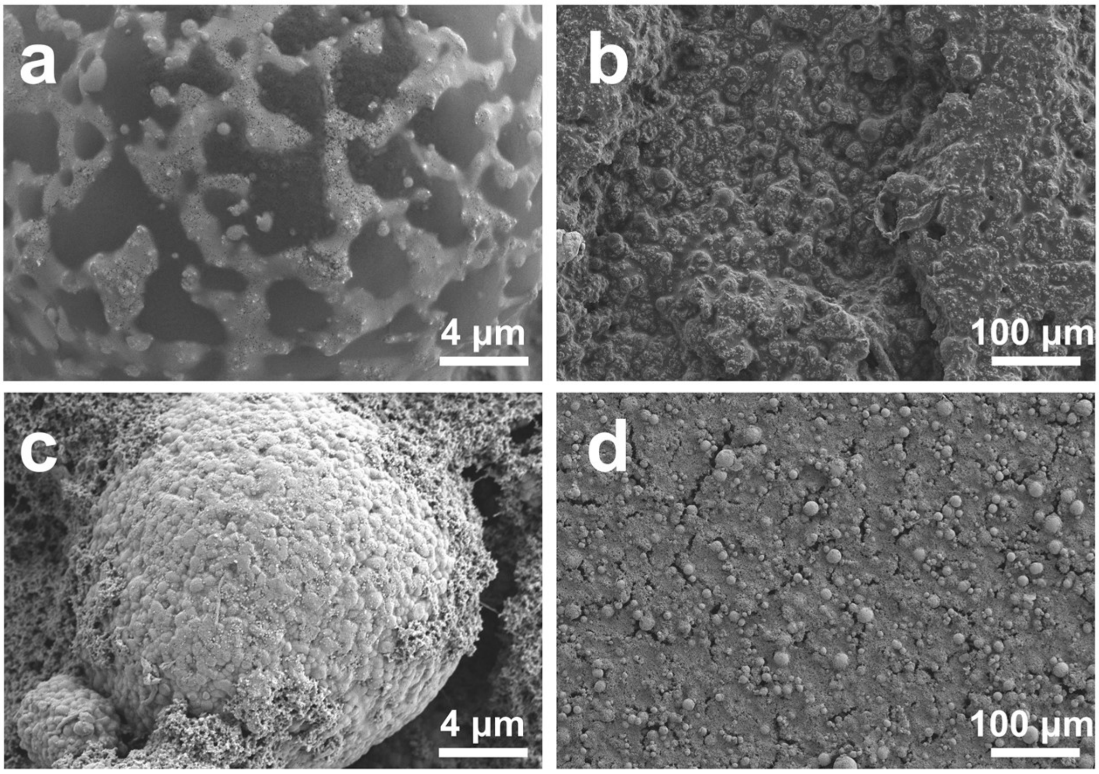

Figure S5. SEM images of cathodes of Li/NCM811(polycrystal) batteries without 2,5BTBA (**a** and **b**) and with 2,5BTBA (**c** and **d**) after 50 cycles at 0.5C.

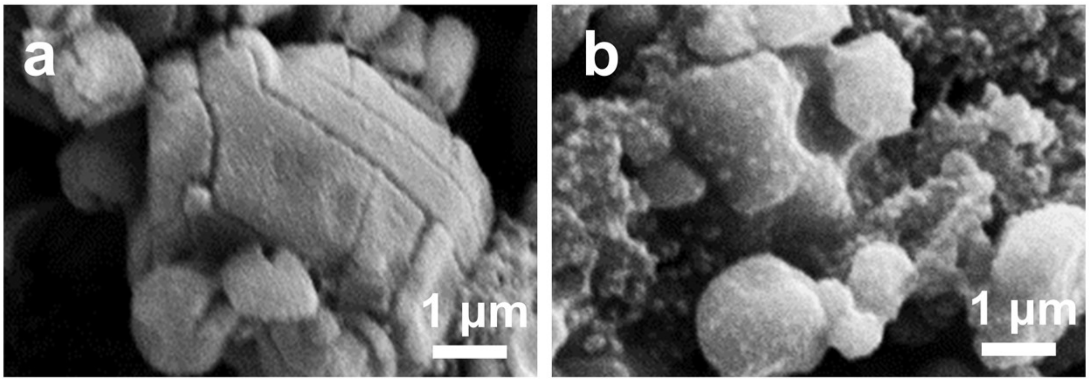

Figure S6. SEM images of cathodes of Li/NCM811(single crystal) batteries without 2,5BTBA (**a**) and with 2,5BTBA (**d**) after 50 cycles at 0.5C.

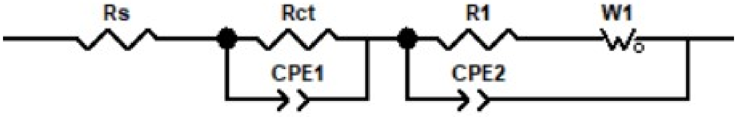

Figure S7. Equivalent circuit models used for EIS spectra in Figure 3e and 3f. The meanings of the symbols are as follows: R_s represents electrolyte resistance, corresponding to intersection of high-frequency semicircle and x axis, R_{ct} represents charge transfer resistance, corresponding to diameter of depressed semicircle, and W_1 represents Warburg impedance of Li^+ diffusion, corresponding to slope line in low-frequency range.

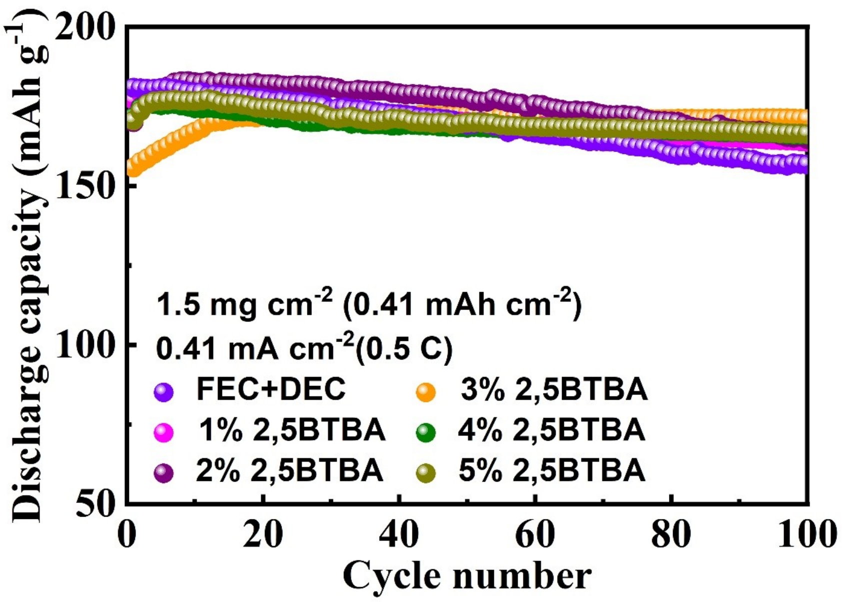

Figure S8. The cycling performance of batteries with different content of additives (1%, 2%, 3%, 4%, and 5% wt. of 2,5BTBA) and without 2,5BTBA.

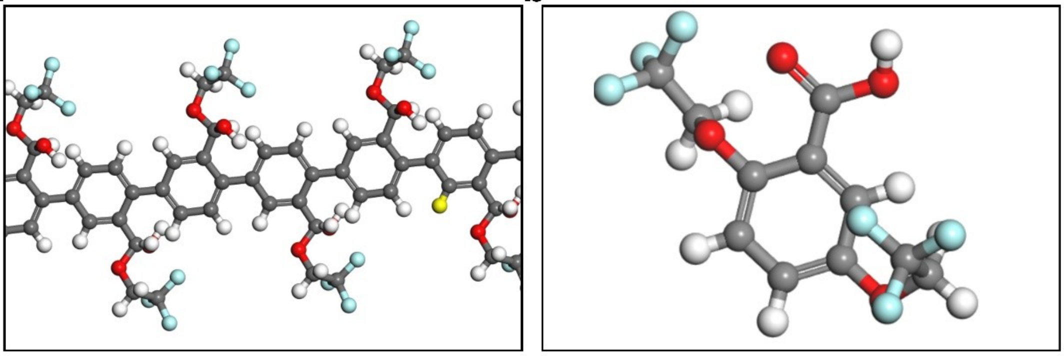

Figure S9. The chemical structure of PTA (**a**) and 2,5BTBA (**b**). Red, white, blue, and gray small balls represent oxygen, hydrogen, fluorine, and carbon atoms, respectively.

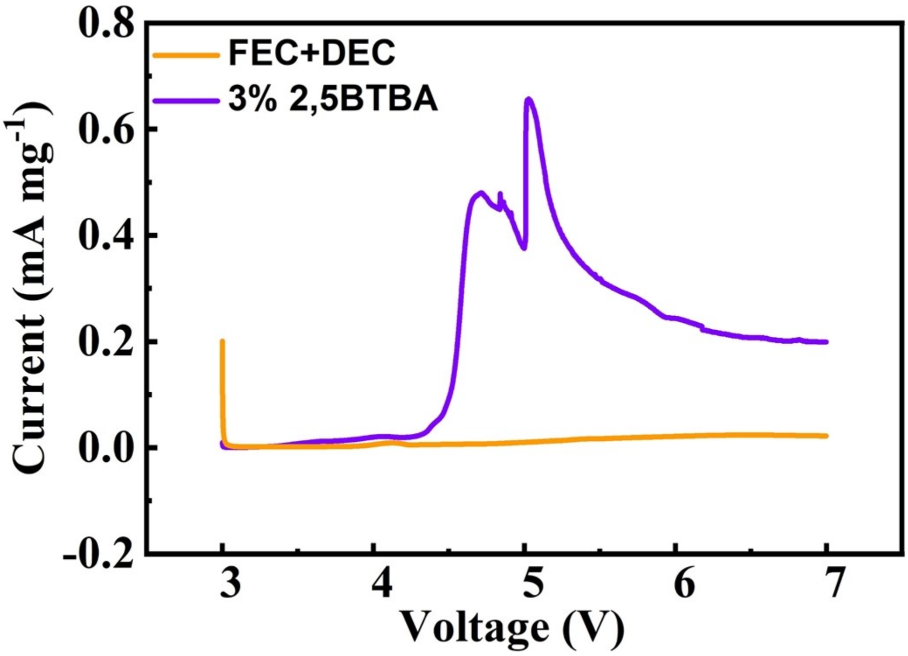

Figure S10. The liner sweep voltammetry of Li/NCM811 batteries without 2,5BTBA and with 2,5BTBA.

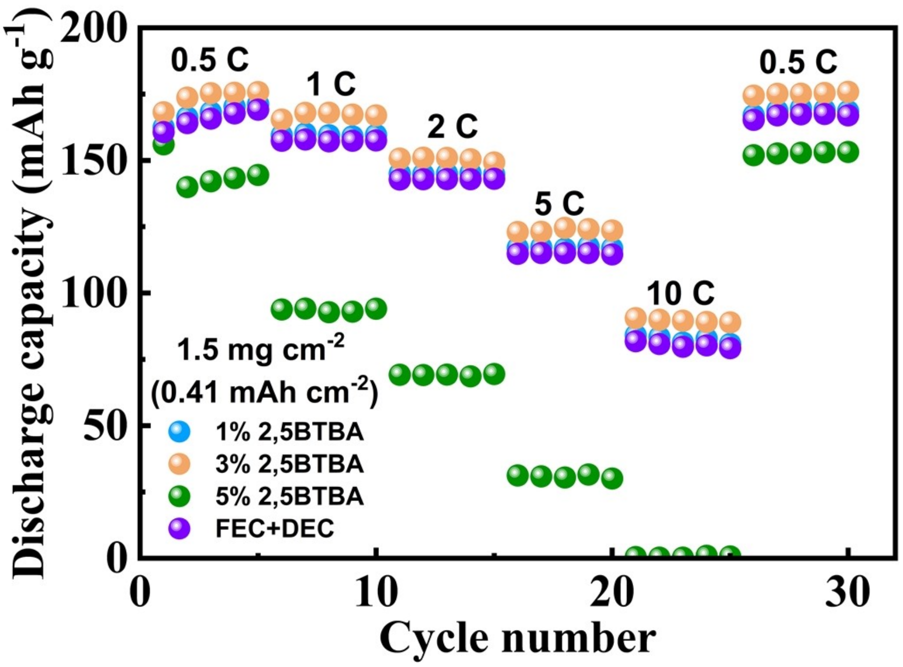

Figure S11. Rate performance of Li/NCM811 batteries with different content of additives (1%, 3% and 5% wt. of 2,5BTBA) and without 2,5BTBA.

Table S1. Fitted results of EIS plot in Figure 3e and 3f.

Samples	Fitted R_s (Ω)	Fitted R_{ct} (Ω)	Fitted R_1 (Ω)
2,5BTBA 50 Cycles	3.634	38.22	64.78
2,5BTBA 100 Cycles	4.431	14.62	45.65
FCE+DEC 50 Cycles	2.722	9.365	28.72
FCE+DEC 100 Cycles	5.462	14.98	31.47
